# Harnessing *Pseudomonas aeruginosa* for Bioremediation: Comparative Study on the Removal of Indigo Carmine and Safranine-T Textile Dyes

**DOI:** 10.1021/acsomega.4c07948

**Published:** 2025-04-09

**Authors:** Magali Teresinha Ritter, Maria Eliza Nagel-Hassemer, Ricardo Mazzon, Amanda Silva Hecktheuer, María Ángeles Lobo-Recio

**Affiliations:** † Department of Environmental Engineering, Federal University of Santa Catarina (UFSC), Campus Reitor João David Ferreira Lima, 88.040-900 Florianópolis, SC, Brazil; ‡ Department of Applied Chemistry, Autonomus University of Madrid (UAM), University City of Cantoblanco, Francisco Tomás y Valiente Street, 2, 28049 Madrid, Spain; § Department of Microbiology, Immunology and Parasitology, UFSC, Campus Reitor João David Ferreira Lima, 88.040-900 Florianópolis, SC, Brazil; ∥ Graduate Programm on Environmental Engineering, UFSC, Campus Reitor João David Ferreira Lima, 88.040-900 Florianópolis, SC, Brazil; ⊥ Department of Energy and Sustainability, UFSC, Campus Araranguá, Rod. Gov. Jorge Lacerda, 3201, Jardim das Avenidas, 88.906-072 Araranguá, SC, Brazil

## Abstract

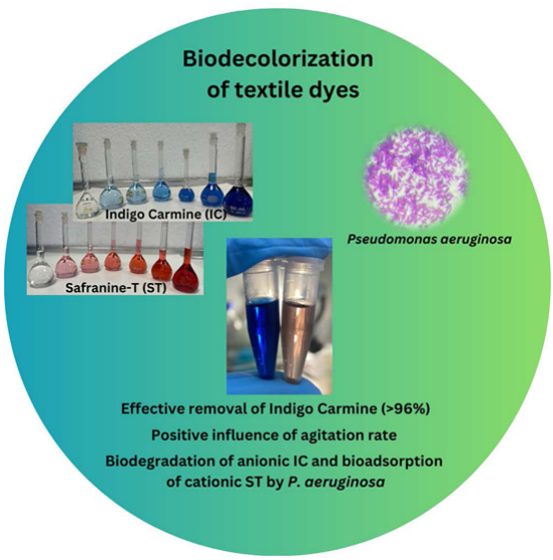

Dyes are a major source of contamination in industrial wastewater, posing significant challenges for effective remediation due to their structural resilience, low biodegradability, recalcitrant behavior, and toxic properties. Given the limitations of conventional treatment methods, recent research has increasingly focused on developing innovative, sustainable, and environmentally friendly solutions to address this pressing issue. This study investigates the potential of *Pseudomonas aeruginosa* in biological remediation by comparing two textile dyes: the anionic indigo carmine (IC) and the cationic safranine-T (ST). The influence of different initial dye concentrations (50, 100, 150, and 500 mg L^–1^), agitation rates (static vs 125 rpm), and growth medium concentrations (25 vs 50 g L^–1^) was also assessed. It was found that *P. aeruginosa* achieved >96% decolorization of IC within 8 h under agitation, with a decolorization rate of 60.27 mg L^–1^ h^–1^. In contrast, ST showed limited removal (77.2%) only in the lowest dye concentration and highest medium concentration. Agitation consistently enhanced dye removal, with IC likely metabolized as an energy source, while ST removal was attributed to biosorption. Second-order kinetic models best described the decolorization process. These findings highlight the potential of *P. aeruginosa* as biological resources for efficient, sustainable wastewater treatment, offering a viable alternative to conventional methods and contributing to environmental sustainability. Further optimization of process conditions could broaden its application in industrial effluent management.

## 1 Introduction

Nowadays, the development of technologies for the decontamination of textile effluents, in order to solve the serious environmental problems arising from their inadequate treatment, is an urgent challenge. As a major player on the world economic scene, the textile industry has a production chain known for generating large volumes of wastewaters with a high polluting potential. Dyes, the most common pollutants in textile effluents, have an annual global production of around 1,000,000 tons, 80% of which are consumed in this sector alone.^1^ It is estimated that approximately 280,000 tons of dyes are lost to the environment every year worldwide, which, along with other chemical used in textile production (dispersants, salts, detergents, and oxidizers), contaminate the soil, sediments, and surface and groundwater, posing a significant global environmental pollution concern.^2,3^

Commercial dyes can be classified in various ways. Based on their application and dyeing mechanism, dyes are categorized as reactive, disperse, direct, acid, and basic dyes.^4^ Considering the chemical structure of the chromophore group responsible for conferring the color, they are identified as azo, anthraquinonic, triphenylmethane, or heterocyclic dyes.^5^ In addition, dyes are also differentiated based on the charge of the particles after dissolving in an aqueous medium, into anionic, cationic, and nonionic (disperse dyes),^6^ which is essential for selecting treatment methods, since processes such as adsorption, coagulation/flocculation, ion exchange, and bioremediation depend directly on the interactions between the dyes and the treatment agents and are strongly affected by the electrical charge of the dyes.^7^

Anionic dyes are highly soluble in water and correspond to all acid dyes, as well as most reactive and direct dyes, representing the main fraction of dyes in wastewater.^8^ With its distinctive blue color, indigo carmine (IC) is one of the most important anionic dyes for the textile industry, especially used to dye jeans.^9^ Around 40,000 tons of IC are produced annually.^10^ The release of unfixed IC dye during textile processing into the environment is extremely dangerous for human health and can permanently affect the corneas and conjunctiva when in direct contact with the eyes, as well as being very harmful to the respiratory tract and causing severe skin irritation. In addition, its carcinogenic nature can lead to neural, developmental, and reproductive disorders.^11^

Cationic dyes, on the other hand, due to the nature of their electrostatic interactions and molecular structure, are less soluble in water and decompose easily when in contact with alkaline substances. They have a high color intensity and are very visible even at low concentrations. In addition, they have more harmful effects compared to anionic dyes, due to their stronger and easier interaction with cell membranes (negatively charged), which can cause skin and mucous membrane irritation and allergic dermatitis.^6,12^ Safranine-T (ST) is one of the oldest known synthetic cationic dyes, widely used in the dyeing of paper, silk, acrylic fibers, leather, and biological analysis procedures.^13^ It is considered one of the model compounds to represent the dyes that are released into the effluents of these industries.^14^ ST is characterized as a nitrogenous heterocyclic dye, which is more difficult to degrade than benzenic compounds, since its degradability is related to properties such as molecular structure, extended aromaticity, particle charge, and physicochemical characteristics.^15^ ST dye can cause several acute health problems, affecting the eyes, lips, mouth, throat, and stomach.^16^

Both cationic and anionic dyes also have harmful effects on the environment, especially causing pollution of water resources, reducing light penetration and impairing photosynthesis, as well as leading to the formation of teratogenic, mutagenic, and carcinogenic byproducts.^17^ Even small concentrations of dyes (10–15 mg L^–1^) in wastewater can significantly affect the transparency, gas solubility, and aesthetic value of watercourses.^18^ Heterocyclic groups (chromophore group), constituents of IC and ST dyes, as well as sulfonic acid (auxochrome group of IC dye) increase solubility and color fastness but provide greater resistance to microbial attack, making them recalcitrant to oxidative degradation.^19^

Due to the low biodegradability and structural stability of most dyes, conventional physicochemical and biological methods, become limited or ineffective.^20^ In coagulation–flocculation processes, the main disadvantage is the generation of large volumes of sludge, which requires neutralization and proper disposal, increasing treatment costs. Filtration involves high electricity consumption and membrane costs. Adsorption, although attractive due to its simple operation and high performance, is highly dependent on the adsorbent materials and can be ineffective for some classes of dyes, especially due to electrostatic interactions between the dye and the surface of the adsorbent. Photocatalysis and electrooxidation processes also involve high energy consumption and high equipment costs,^21^ while Advanced Oxidative Processes (AOPs), although efficient, can increase the toxicity of treated wastewater and are therefore considered an environmentally unfriendly technology.^22^ To overcome these shortcomings, the current trend is toward efficient, economical, and eco-friendly remediation technologies, preferably developed from renewable and sustainable energy sources, using biotechnology as a possible solution.^23^

In this sense, biological remediation are considered a promising alternative to other existing treatment technologies, as they fulfill all these requirements, being more attractive, less expensive, and beneficial to the environment.^21,24^ Many microorganisms have been tested to decolorize textile dyes, mostly anaerobic bacteria.^25^ However, it has been reported that the anaerobic biodecolorization process can lead to the formation of aromatic amines, which are potentially mutagenic and carcinogenic, and therefore, the use of aerobic bacterial strains should be considered.^19^ Among them, *Pseudomonas aeruginosa* stands out as one of the few capable of degrading dyes aerobically and is one of the dominant species present in the sludge of textile wastewater treatment plants, capable of surviving in aerobic and anoxic environments and competing in complex ecosystems with substrate limitations.^26−28^ Its versatile metabolism and applications in waste treatment, as well as in the production of compounds such as antibiotics and enzymes, have awakened considerable interest.^29^ Moreover, several studies have shown that *P. aeruginosa* had the greatest ability to degrade different dyes compared to the other bacterial strains tested.^15,18,24,30−34^

Considering this, the present work aims to compare the biodecolorization efficiency of the indigo carmine and safranine-T dyes by *P. aeruginosa*. To the best of our knowledge, studies reporting the biological degradation of these two dyes separately are scarce and no data has been published regarding the biodecolorization process comparing these anionic and cationic dyes. Furthermore, the influence of different dye concentrations, agitation rates, and nutritional concentrations in the growth medium on the bioremoval process of textile dyes was studied. The kinetic study for the samples with the best removals of both dyes was also carried out. Understanding the differences in the decolorization process of anionic and cationic dyes using microorganisms such as *P. aeruginosa* is crucial for developing and enhancing wastewater treatment technologies. While combining or coupling technologies to improve dye removal performance and reduce costs is necessary, evaluating the performance of treatments on different types of dyes is fundamental to developing versatile, efficient, and sustainable systems that meet the demands of the textile sector and environmental standards.

## 2 Material and Methods

### 2.1 Textile Dyes

IC and ST dyes were purchased from Neon Comercial (Brazil). IC P.A. (CI. 73015), also known as Acid Blue 74, was used as a representative of anionic dyes, while ST P.A. (CI. 50240) is a cationic dye, also called Basic Red 2.

The dye solutions were prepared by diluting the stock solution (1 g L^–1^ in distilled water) in a Phosphate Buffer Solution (PBS). The PBS has the following composition: 8.0 g L^–1^ sodium chloride (NaCl), 1.44 g L^–1^ disodium phosphate (Na_2_HPO_4_), 0.24 g L^–1^ monopotassium phosphate (KH_2_PO_4_), and 0.2 g L^–1^ potassium chloride (KCl). The aqueous solutions were sterilized by filtration through sterile 0.22 μm pore size filters, following the procedure proposed by Behzat.^35^

### 2.2 Strain and Growth Conditions

A pure culture of *P. aeruginosa* (strain ATCC 27853) was used as the inoculum in this study. The bacteria were grown overnight on a nutrient agar plate incubated at 37 ± 1 °C. The inoculum was routinely prepared by dispersing a colony of bacterial cells collected from the agar plate in the Luria–Bertani (LB) broth (KASVI, Brazil) and incubated at 37 ± 1 °C with constant stirring (125 rpm) until it reached an Optical Density (O.D.) between 0.6 and 0.8, measured in a HACH DR/3900 ultraviolet–visible (UV–vis) spectrophotometer with a selected wavelength of 625 nm. This optical density range corresponds to the logarithmic phase of bacterial growth on the McFarland scale, in which around 1.5 × 10^8^ cells mL^–1^ are estimated.^36^ Subsequently, the required volumes of bacterial suspensions were added to the LB broth in order to obtain an O.D. of 0.1.^37^ The composition of the LB broth was sodium chloride (NaCl, 10 g L^–1^), tryptone (10 g L^–1^), and yeast extract (5 g L^–1^). The reaction medium used in the biodecolorization tests was prepared (1:1 volume ratio) with the bacterial suspension in LB broth and dye solutions (in the required concentrations) buffered in PBS. All the glassware and solutions were sterilized in a Luferco model 39211 vertical autoclave at 121 ± 1 °C for 15 min.

### 2.3 Biodecolorization Experiments

Biodecolorization tests were conducted to evaluate the influence of different parameters, including the type and concentration of dyes, agitation rate, and concentration of LB culture medium on the removal of IC and ST dyes by *P. aeruginosa*.

The assays were carried out in 250 mL Erlenmeyer flasks, with 120 mL of reaction medium, half the volume consisting of bacterial culture in LB broth and the other half of dye solution at the required concentration (buffered in PBS). The experiments were conducted at room temperature using an orbital shaker (Kasvi K40–3020). The pH was monitored throughout the experiments and remained neutral, with values between 7 and 7.2. All experiments were carried out in duplicate and the results reported as the average value obtained. Controls without microbial culture were also performed.

Different dye types (anionic and cationic) and concentrations (50, 100, 150, and 500 mg L^–1^) were studied, as well as the effect of the agitation rate (125 rpm and static condition) and the concentration of nutrients in the growth medium (LB broth - KASVI) (25 and 50 g L^–1^) on the biodecolorization of the textile dyes. Samples were labeled LBA, 2LBA, LBS, and 2LBS, where LB refers to the medium with 25 g L^–1^ and 2LB for the doubled concentration of 50 g L^–1^, under agitation (A) or static (S) condition.

The biodecolorization tests were carried out over 48 h, with samples collected at time intervals of 0.5, 1, 2, 3, 4, 8, 12, 24, and 48 h. After treatment, the samples were collected (sterile conditions) and centrifuged at 4000 rpm for 10 min. The final concentration of the dyes in the supernatant was determined by colorimetry from calibration curves (concentration range 10 to 125 mg L^–1^) at wavelengths of 610 and 520 nm for IC an ST, respectively, in a HACH DR/3900 spectrophotometer. The bioremoval efficiency *R* (%) was calculated using eq 1, where *C*_i_ and *C_t_* are the initial and at the time *t* (min) dye concentrations (mg L^–1^), respectively.
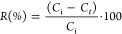
1

The biodecolorization rate (*B*_R_, mg L^–1^ h^–1^), which refers to the speed with which the dye removal process occurs, was calculated according to eq 2, where *C*_f_ is the final dye concentration (mg L^–1^).
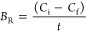
2

A kinetics study was conducted for the tests with the highest removals of each dye. The zero, first, and second-order linear equations used are shown in Table 1.

**Table 1:** Equations at Different Orders (*n*) to Determine the Decay of the Indigo Carmine (IC) and Safranine-T (ST) Dyes (Adapted from Behzat^35^)

order (*n*)	linearized equation	plot	*K*_*n*_ unit
**0**	*C_t_* = *C*_i_ – *K*_0_*t*	*C_t_* versus *t*	mg L^–1^ h^–1^
**1**	ln *C*_t_ = ln *C_i_* – *K*_1_*t*	ln *C_t_* versus *t*	h^–1^
**2**	1/*C_t_* = 1/*C*_i_ + K_2_*t*	1/*C_t_* versus *t*	L mg^–1^ h^–1^

### 2.4 Statistical Analysis

STATISTICA 13.3 software was used to evaluate the effects of the different parameters on the biodecolorization of textile dyes. ANOVA was applied to test for differences between the means of data with normal distribution, followed by Tukey’s test. Differences were considered significant when *p* < 0.05.

## 3 Results and Discussion

### 3.1 Effect of the Initial Dye Concentration, Growth Medium Concentration, and Agitation Rate on the Biodecolorization of Textile Dyes

**Figure 1:**
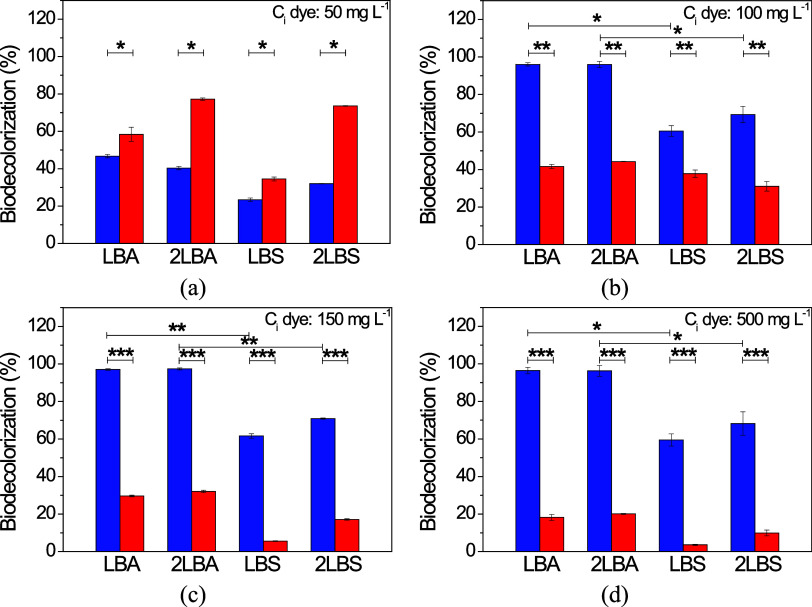
Effect of growth medium and agitation on the biodecolorization at different initial dye concentrations: (a) 50 mg L^–1^, (b) 100 mg L^–1^, (c) 150 mg L^–1^, and (d) 500 mg L^–1^ of indigo carmine (in blue) and safranine-T (in red) by *P. aeruginosa*. Asterisks indicate statistically different means according to Tukey’s test (*p*-value <0.05*; < 0.005**; < 0.0005***).

The results of the biodecolorization by *P. aeruginosa*, comparing the IC and ST dyes over the 48 h test are shown in Figure 1 and Table 2.

**Table 2:** Quantification and Bioremoval Efficiencies of Indigo Carmine (IC) and Safranine-T (ST) Dyes by *P. aeruginosa* under Different Experimental Conditions

indigo carmine								
samples	LBA		2LBA		LBS		2LBS	
*C*_*i*_ (mg L^–1^)^a^	*R* (%)^b^	*Q* (mg L^–1^)^c^	*R* (%)	*Q* (mg L^–1^)	*R* (%)	*Q* (mg L^–1^)	*R* (%)	*Q* (mg L^–1^)
50	46.7	23.36 ± 0.94	40.3	20.17 ± 0.87	23.4	11.70 ± 0.93	32.0	16.00 ± 0.15
100	96.0	96.03 ± 0.82	96.0	96.00 ± 1.65	60.5	60.54 ± 2.86	69.3	69.31 ± 4.35
150	97.0	145.6 ± 0.43	97.4	146.0 ± 0.53	61.6	92.40 ± 1.20	70.9	106.4 ± 0.30
500	96.4	482.2 ± 1.77	96.3	481.4 ± 2.89	59.5	297.5 ± 3.25	68.2	341.0 ± 6.25
safranine-T								
50	58.4	29.19 ± 3.82	77.2	38.61 ± 0.66	34.5	17.24 ± 1.07	73.6	36.80 ± 0.17
100	41.6	41.59 ± 1.07	44.2	44.25 ± 0.13	37.8	37.82 ± 1.94	31.0	31.03 ± 2.53
150	29.7	44.49 ± 0.42	32.0	48.07 ± 0.64	5.6	8.43 ± 0.21	17.1	25.73 ± 0.41
500	18.2	91.15 ± 1.58	20.1	100.5 ± 0.27	3.6	18.20 ± 0.35	9.9	49.65 ± 1.58

a *C*_i_: initial dye concentration (mg L^–1^).

b *R* (%): bioremoval efficiency (%).

c *Q*: quantitative removal (mg L^–1^).

From Figure 1, it can be seen that, with the exception of the lowest concentration (50 mg L^–1^) at which ST showed the best results, the bioremoval performance of the IC dye was superior for all the other initial dye concentrations tested. When using 50 mg L^–1^ (Figure 1a), the maximum removal of IC (46.7%–23.4 mg L^–1^) occurred in the LBA condition (under agitation). For ST, bioremoval reached 73.6% (36.8 mg L^–1^) and 77.2% (38.6 mg L^–1^) for 2LBS and 2LBA, respectively. Applying a concentration of 100 mg L^–1^ (Figure 1b), IC bioremoval was very high, reaching 96.0% efficiency under agitation (LBA and 2LBA). With ST, on the other hand, bioremoval efficiencies did not exceed 45% (2LBA). At this concentration, as with the IC dye, the ST samples with the best removal percentages were those under stirring conditions. The biodecolorization behavior of the dyes was similar to the previous one when an initial dye concentration of 150 mg L^–1^ was used (Figure 1c). Bioremoval of the IC dye reached over 97.0% (>145 mg L^–1^) for both, LBA and 2LBA. The best performing sample for ST dye was 2LBA, which achieved approximately 32.0% (48.1 mg L^–1^) removal efficiency. Finally, a concentration of 500 mg L^–1^ (Figure 1d) was used to assess the ability of *P. aeruginosa* to degrade solutions considered to have high dye loads, since according to Singh,^38^ the concentration of dyes ranges from 10 to 200 mg L^–1^ in textile effluents. For ST, unimpressive results were obtained, 20.1% (2LBA), 18.2% (LBA), 9.9% (2LBS), and 3.6% (LBS). However, the biodecolorization of the IC dye achieved significant removal efficiencies, even at this very high concentration. Both LBA and 2LBA showed over 96.0% (>480 mg L^–1^) removal efficiency at the end of the experiment. In short, while the decolorization of ST decreased significantly with an increase in its initial concentration, raising the initial concentration of IC, even at high dye loads, led to its effective biodecolorization, when under agitation. This trend suggests that different mechanisms are involved in the bacterial decolorization processes for IC and ST.

Statistical analysis showed that the parameters that significantly influenced (*p* < 0.05) the biodecolorization of the dyes were the dye type and agitation rate. It was found that the concentration of the growth medium (LB or 2LB) had no significant effect on the dye bioremoval process. This was evidenced by the bacterial growth curves with similar colony-forming units for the two nutritional conditions tested (data not shown). The two main mechanisms involved in the biological decolorization of dyes are biodegradation, in which the dyes are metabolized through enzymatic action, and biosorption, in which the dyes are adsorbed under the bacterial cell walls.^39^ In the latter, the molecular structure of the dye and the electrical charges on the surface of the bacterial biomass have a crucial effect.^40^*P. aeruginosa* is classified as a Gram-negative bacterium, so when subjected to Gram staining and treated with different reagents, it acquires a reddish color (conferred by the safranine-T dye applied in this technique), unlike Gram-positive bacteria, which turn purple.^41^ Due to the structure and composition of their cell walls and outer membrane, made up predominantly of phospholipids and lipopolysaccharides, their surface electrical charge is negative.^42^ Thus, considering the negatively charged surface of the bacterial biomass and the cationic form of the dye in solution (positive), it can be inferred that the bacterial membrane is capable of attaching the safranine-T molecules, even though it does not metabolize them, and that the dye removal was therefore due to the biosorption mechanism. In this way, the scarce removal of ST observed in the tests can be attributed to the dye adhering to the bacterial membranes, explaining why the percentage of decolorization decreases when the concentration of safranine increases since the initial bacterial biomass was similar in all the experiments. The reddish color observed in the pellet formed after centrifuging the samples is an indication that corroborates this statement.

In contrast to ST, the biosorption of negatively charged particles of the anionic dye indigo carmine in solution was not favored by the also negative surface charge of *P. aeruginosa*, considering that there was no change in the pH of the reaction medium. Srinivasan et al.^40^ highlighted the influence of pH on the biosorption process, suggesting that the use of an acid pretreatment could change the surface charges of the bacterial biomass to positive, increasing its attraction to the anionic dye. However, in this study, the pH remained between 7 and 7.2 during all the tests, which rules out potential modifications in surface charge interactions. Meanwhile, Boran et al.^30^ tested live and dead pellets of *P. aeruginosa* and showed that the biodecolorization of the IC dye was mainly due to bacterial metabolism and not to the adsorption process. The poor removal efficiency at the lowest dye concentration also indicates that IC degradation occurs via enzymatic activity, as it is common in the bacterial regulatory mechanism for the expression of many enzymes to be modulated by the presence of the substrate on which they will act, and low dye concentrations may not be sufficient to induce this response.^39^

With regard to agitation, it was a key factor in the biodecolorization of IC and ST dyes, leading to higher removal efficiencies in all the conditions evaluated. Ramya et al.^43^ state that the increase in discoloration in a culture kept under agitation can be attributed to an increase in the activity of microbial enzymes. Agitation is also considered a factor that positively influences the adsorption process, increasing the transfer of external mass.^44^ This may explain why, under static conditions, it was not possible to establish a pattern between the amount of ST removed and the increase in the initial concentration of the dye, since the exposed surface of the bacteria in this condition may not have been the same, preventing biosorption from occurring homogeneously as when under agitation. In this regard, dye removal was favored under shaking conditions, regardless of the decolorization mechanism, whether via biosorption for ST or via biodegradation for IC.

These findings contribute valuable insights to the field of bioremediation, particularly regarding the mechanisms involved in dye removal by *P. aeruginosa*. The removal of ST via biosorption rather than biodegradation is supported by previous studies highlighting the role of bacterial surface charge in cationic dye adsorption.^12^ In addition, the reddish hue of the bacterial pellet and the stability of the pH, which excludes possible changes in electrostatic interactions, corroborate this approach. However, the results further emphasize that biosorption alone may not be a viable long-term strategy for complete dye degradation. In contrast, the behavior observed for IC further reinforces that its degradation is enzyme-mediated, since bacterial metabolic pathways are often regulated by substrate availability. Higher removal efficiencies with increasing IC initial concentration demonstrate that the greater substrate availability likely triggered a stronger metabolic response. Likewise, previous reports also support that bacterial metabolism plays a crucial role in the bioremediation of anionic dyes.^30,31^ The observed dependency on dye concentration highlights the necessity of optimizing operational parameters to ensure effective enzyme induction, a factor often overlooked in similar studies. Additionally, the significant impact of agitation on removal mechanisms underscores its importance as a key operational parameter for scaling up bioremediation processes.

### 3.2 Kinetic Studies on the Biodecolorization of Textile Dyes

**Figure 2:**
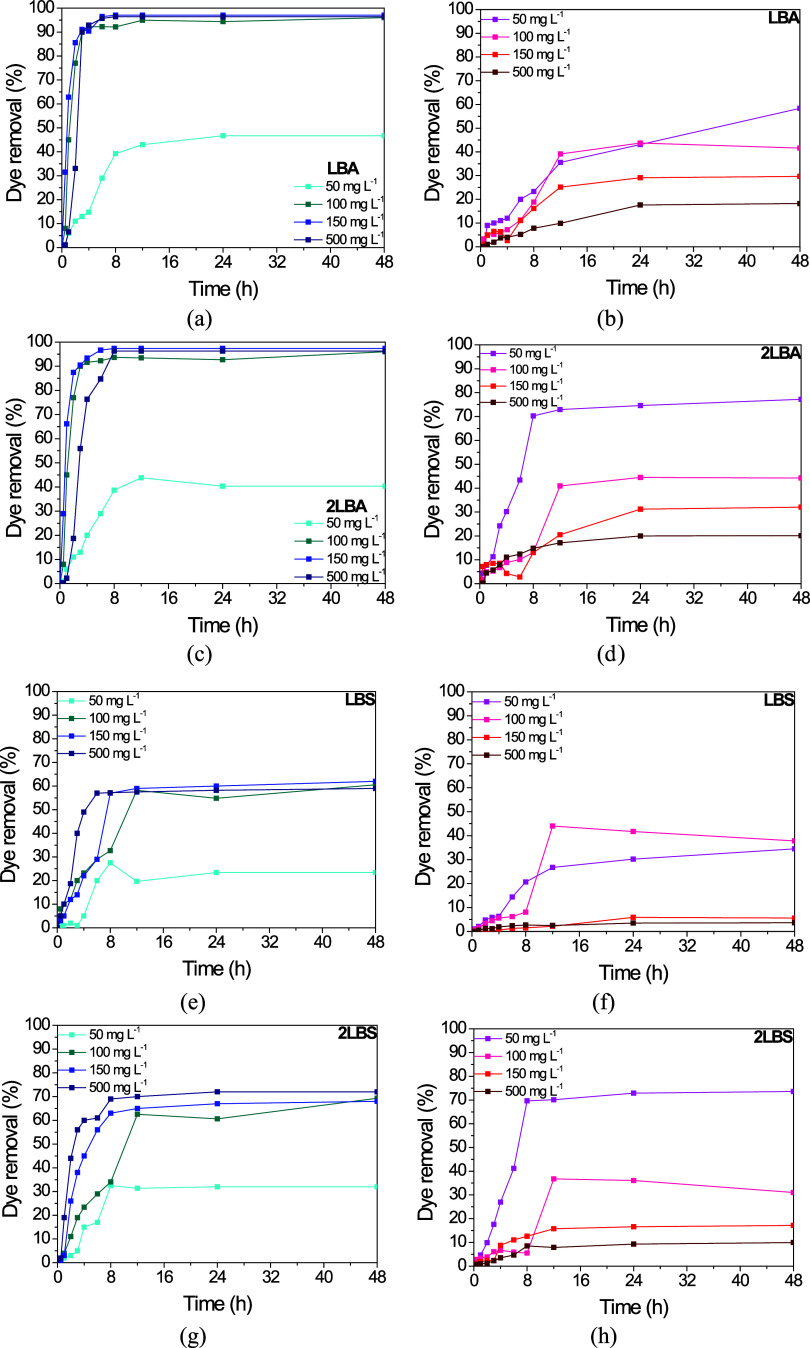
Biodecolorizaton kinetics at different initial concentration of (a, c, e, g) indigo carmine and (b, d, f, h) safranine-T dyes by *P. aeruginosa*.

The initial concentration of dyes has an important effect on the decolorization process, since high concentrations can negatively affect the efficiency of the biodecolorization due to possible toxicity to microorganisms.^38^ The IC and ST biodecolorization tests were conducted using different initial dye concentrations 50, 100, 150, and 500 mg L^–1^. Figure 2 shows the biodecolorization kinetics of the two dyes at these different concentrations as a function of time.

The results obtained with the IC showed that higher removal efficiencies were achieved as the initial concentration of the dye increased (Figure 2). When an initial dye concentration of 50 mg L^–1^ was applied, the bioremoval of the dye by *P. aeruginosa* did not exceed 50% in any of the conditions evaluated, but as the concentration increased to 100, 150, and 500 mg L^–1^, practically quantitative removals (>96.0%) were obtained for the samples kept under agitation. These findings are superior to those reported by most authors on the biodegradation of this dye using different bacteria (Table 3). Oh et al.^45^ using *Citrobacter amalonaticus* Y19 obtained a biodecolorization of only 12.5% when applying a low initial concentration of dye (50 mg L^–1^). *Pseudomonas* strains, Z1, GM3, and Q3, used by Yu et al.^46^ at 48 h incubation time and 100 mg L^–1^ initial dye concentration, did not exceed removal efficiencies of 88, 69, and 61%, respectively. Boran et al.^30^ were able to obtain a high IC removal efficiency of 92% using an initial concentration of 50 mg L^–1^. However, the stirring rate and temperature used were slightly higher. Furthermore, when they tested a high dye load (500 mg L^–1^), the bioremoval efficiency was approximately 60% (8 h), considerably lower when compared to the 96.4% obtained in this study for the same period of time.

**Table 3:** Indigo Carmine Biodecolorization by Different Bacteria

bacteria	*C_i_* (mg L^–1^)^a^	*t* (h)^b^	*T* (°C)^c^	agitation (rpm)	*R* (%)^d^	refs
*Citrobacter amalonaticus* Y19	50	48	35	100	12.5	(45)
*Bacillus sp*.	100	96	37	120	66.6	(49)
*Trametes trogiiTrametes villosaCoriolus versicolor*	23.4	24	28	static	95 93 95	(50)
*Paenibacillus larvae*	100	8	30	150	100	(43)
*Bacillus sp*. *MZS10*	100	15	30	stirred	87.31	(51)
*Bacillus aryabhattai* DC100	180	72	37	304.09	98.31	(52)
*Aeromonas hydrophila* DEC1	100	24	30	static	60	(53)
*Pseudomonas sp*. *Z1 Pseudomonas sp*. *GM3 Pseudomonas sp*. *Q3*	100	48	35	static	88 69 61	(46)
*Pseudomonas aeruginosa* ATCC 10145	50	6	30 40	150	92 93	(30)
*Pseudomonas aeruginosa* ATCC 27853	50 100 150 500	8	23	125	39.2 92.2 97.4 96.4	this study
*Pseudomonas aeruginosa* ATCC 27853	50 100 150 500	48	23	125	46.7 96.0 97.4 96.4	this study
*Pseudomonas aeruginosa* ATCC 27853	50 100 150 500	48	23	static	23.4 60.5 61.6 59.5	this study

a *C_i_*: initial dye concentration (mg L^–1^).

b *t*: time (h).

c *T*: temperature (°C).

d *R*(%): bioremoval efficiency (%).

Even when compared to other treatment technologies, such as adsorption and AOPs, recognized for their effectiveness in color removal, the biological remediation of IC by *P. aeruginosa* in this study demonstrated superior results. Using AOPs, Castillo-Suárez^21^ reported high IC dye removal efficiencies (82–100%), and very fast treatment times, from 25 to 90 min, but this performance was only achieved at low dye concentrations (≤25 mg L^–1^). At higher dye concentrations (500 mg L^–1^), IC adsorption using nanoporous magnesium aluminophosphate, at a dosage of 2.5 g L^–1^, a temperature of 30 °C and a contact time of 20 min did not exceed 35%.^47^ With an initial concentration of 600 mg L^–1^, Terminalia Catappa bark biosorbent achieved 73% efficiency at 25 °C in 30 min, but required pH 2.^48^

As shown in Figure 2, for a dye concentration of 500 mg L^–1^, it was observed that the effective removal of IC occurred mainly in the first 8 h of the test, regardless of the initial concentration used. However, except for the lowest concentration (50 mg L^–1^), bioremovals of over 90% were achieved within the first 3 h for the LBA and 2LBA samples. A different behavior was observed when a concentration of 100 mg L^–1^ was used in the static assays (LBS and 2LBS), which required 12 h to achieve maximum dye biodegradation. In addition to the high removal efficiencies, high biodecolorization rates were also observed (Table 4). For the best conditions (LBA and 2LBA), increasing the IC concentration from 50 to 500 mg L^–1^ led to a faster removal process, ranging from 2.45 to 60.27 mg L^–1^ h^–1^, respectively. The latter is one of the highest removal rates obtained for IC biodecolorization. Previously mentioned studies have reported decolorization rates ranging from 0.13 to 1.44 mg L^–1^ h^–1^^45,46^ and reaching a maximum of 37.5 mg L^–1^ h^–1^ when high IC dye loads were applied.^30^ The results of this study highlight the remarkable ability of the selected bacterial strain to biodecolorize the anionic dye IC under moderate conditions of time, temperature, pH and agitation, which greatly favors its practical application.

**Table 4:** Biodecolorization Rates Obtained using Different Initial Concentrations of the Dyes Indigo Carmine (8 hours test) and Safranine-T (24-hours test)

biodecolorization rate (mg L^–1^ h^–1^)								
	indigo carmine				safranine-T			
	dye concentration (mg L^–1^)							
samples	50	100	150	500	50	100	150	500
LBA	2.45	11.52	18.20	60.27	0.90	1.82	1.82	3.67
2LBA	2.42	11.71	18.26	60.17	1.55	1.85	1.95	4.17
LBS	1.72	4.08	10.69	35.75	0.63	1.74	0.37	0.73
2LBS	2.03	4.26	12.94	39.38	1.52	1.50	1.04	1.94

In the case of ST dye, increasing the initial dye concentration led to a decrease in removal efficiency, although similar amounts (in mg L^–1^) were biosorbed at concentrations of 50 to 150 mg L^–1^ (Table 2). The removal kinetics indicated that the maximum biosorption of ST by *P. aeruginosa* was reached in 24 h of testing, after which some of the samples showed a slight decline in removal efficiency, attributed to a possible desorption process of the dye. ST dye also showed lower bioremoval rates than the anionic one, ranging from 1.55 to 4.17 mg L^–1^ h^–1^ (sample 2LBA) at concentrations of 50 and 500 mg L^–1^, respectively (Table 4). Furthermore, for the static samples, the bioremoval rates did not show a linear behavior as occurred with the stirred samples or with the IC dye, since with the increase in the intermediate concentration from 100 to 150 mg L^–1^, the biodecolorization rates decreased. As previously mentioned, however, this is probably due to differences in the external mass transfer process, the first step in the adsorption mechanism, which is strongly influenced by agitation and the consequent available surface area of the bacterial biomass.

Table 5 shows the kinetic constants calculated using linear regression for the samples with the best bioremovals of each dye by *P. aeruginosa*. In this sense, for the IC dye, the kinetic study (8 h) was carried out for the LBA sample at the two highest concentrations. For ST, the kinetic behavior (24 h) of the 2LBA sample was evaluated, which showed the best removal efficiency at 50 mg L^–1^ (77.2%) and the highest amount removed (100.5 mg L^–1^) at 500 mg L^–1^.

**Table 5:** Kinetic Rate Constants Obtained in the Best Bioremoval Conditions for Indigo Carmine (IC) and Safranine-T (ST) Dyes

dye		indigo carmine		safranine-T	
reaction time		8 h		24 h	
dye concentration (mg L^–1^)		150	500	50	500
order (*n*)	rate constants	LBA	LBA	2LBA	2LBA
0	*K*_0_ (mg L^–1^ h^–1^)	14.682	71.298	1.717	4.075
	*R*^2^	0.5898	0.7625	0.7216	0.7590
1	*K*_1_ (h^–1^)	0.439	0.498	0.066	0.009
	*R*^2^	0.8888	0.8659	0.7667	0.7837
2	*K*_2_ (L mg^–1^ h^–1^)	0.029	0.008	0.003	2 × 10^–05^
	*R*^2^	0.9523	0.9587	0.7952	0.8076

The comparison between the coefficients of determination (*R*^2^) of the different kinetic orders showed that the biodecolorization of IC can be better explained by second-order kinetics at both concentrations. The *R*^2^ were in the range of 0.9523 and 0.9587 for the concentrations of 150 and 500 mg L^–1^, respectively. This assumes that the decolorization rate increases quadratically as the dye concentration increases, which is in line with the removal behavior observed for this dye. The results obtained for ST also showed a better fit for second-order kinetics, although the coefficients of determination were quite low (*R*^2^ = 0.7952 and 0.8076). Second-order kinetics for the removal of dye (Mordant Yellow 10) by *P. aeruginosa* was also reported by Rethinam et al.^54^

The results found in this study showed that the *P. aeruginosa* behaved quite differently when bioremoving the different dyes used. While indigo carmine was effectively decolorized in practically all the concentrations tested (100, 150, and 500 mg L^–1^), safranine-T dye showed a satisfactory percentual result (77.2%) only when using the lowest dye concentration (50 mg L^–1^), and its highest quantitative removal (100.5 mg L^–1^) occurred at 500 mg L^–1^. In this case, considering that the uptake of ST is due to its affinity with bacterial cell structures, this behavior can be explained by the driving force generated by the high concentration of dye ions required to overcome the mass transfer between the solid and liquid phases.^55^

Although various bacterial strains have been shown to cometabolize dyes in the presence of carbon and nitrogen sources, *Pseudomonas* are among the very few that have the ability to use dyes as their sole source of energy.^56^ Stolz^57^ reported that *Pseudomonas* enzymes are capable of reductively cleaving sulfonated structures (present in IC) in addition to the carboxylate groups present in different substrates (glucose, acetate, LB broth).

Furthermore, several studies have shown, through the analysis of metabolites, that the degradation mechanisms can differ completely depending, in addition to the added carbon source, on the bacterial strain and the molecular structure of the dye.^25,51^ Generally, these degradation processes involve oxidation, reduction, hydrolysis, and decarboxylation in multiple steps.^58^ In the cyanobacterium *Phormidium autumnale* UTEX 1580, this process can take up to 16 days, involving the production of the secondary metabolite isatin, which ultimately leads to the formation of anthranilic acid. The first step in this transformation involves the oxidative cleavage of the central bond, generating isatin, which is subsequently hydrolyzed, reduced and decarboxylated to form anthranilic acid.^59^

Campos et al.^60^ demonstrated, through in vitro assays using purified laccases from the fungi *Trametes hirsuta* and *Sclerotium rolfsii*, that the stoichiometric ratio of molecular oxygen consumption in the oxidation reaction of IC is 1:1, occurring in two steps. These reactions can be carried out by the laccase in the absence of another redox-active component. However, once isatin is formed, its hydrolysis and decarboxylation to generate anthranilic acid occur independently of the laccase activity.

In *Bacillus safensis* HL3, three enzymes were described as participants in the degradation of IC: a laccase (a copper-dependent multicomponent polyphenol oxidase), an intracellular tyrosinase (l-tyrosine oxidase), and a lignin peroxidase (an oxidase that uses hydrogen peroxide to degrade lignin).^61^ In *Escherichia coli* O157:H7, Sakai strain, a peroxidase from the Dyp family, named YfeX, has been described as being involved in the decomposition of dyes.^62^ This family of peroxidases has also been reported in the fungus *Geotrichum candidum* as capable of decolorizing a variety of dyes, including Reactive Blue 5, Reactive Blue 19, Reactive Blue 114, AQ-1, AQ-2, AQ-2d, Reactive Black 5, Reactive Red 33, Reactive Yellow 2 and RB182.^63^

The genome of *P. aeruginosa* ATCC 27853 contains numerous genes encoding proteins similar to those described for the aforementioned microorganisms, such as oxidases, hydrolases and peroxidases. Among the nine putative peroxidases annotated in the genome of this bacterium, the one encoded by locus ACG06_11210 stands out, being a peroxidase (1.11.1.19) from the Dyp family, as described by Kim and Shoda,^63^ Sugano,^64^ and Liu et al.^62^ Additionally, two genes ACG06_19880 and ACG06_26245, encoding putative copper-dependent polyphenol oxidases are noteworthy, which may be components of a laccase (1.10.3.2) in this organism.

It is therefore possible that the initial stages of IC degradation to isatin are facilitated by some of these enzymes present in *P. aeruginosa*, which have substantial support in the literature regarding their involvement in the oxidative step of the process. However, little can be conjectured about the subsequent steps involving hydrolysis and decarboxylation, culminating in the synthesis of anthranilate. In the work by Wang et al.,^61^ the authors propose that the transformation of isatin to 2-aminophenylacetic acid occurs in two steps, involving hydrolysis and hydration with the loss of one molecular oxygen, followed by decarboxylation to ultimately produce anthranilate. In *P. aeruginosa*, anthranilate is an intermediate in the tryptophan metabolism pathway (KEGG pathway 00380), being produced by the enzyme kynureninase (3.7.1.3) from l-kynurenine, which in turn is derived through the activity of the enzyme arylformamidase (3.5.1.9) using *N*-formylkynurenine as a substrate. Another possible route for anthranilate production is through the activity of the arylformamidase enzyme using formylanthranilate as a substrate, a compound obtained from *N*-formylkynurenine by the action of kynureninase. *N*-formylkynurenine, in turn, is derived directly from tryptophan via the activity of the enzyme tryptophan 2,3-dioxygenase (1.13.11.11). The comparison of the structures of isatin and 2-aminophenylacetic acid with the intermediates in the anthranilate biosynthesis pathway, as well as the evaluation of substrate recognition specificity by kynureninase and arylformamidase, may provide insight into the hydrolysis and decarboxylation steps of the IC degradation pathway in this organism, in future studies.

Although reasonably explained on the basis of the data obtained and the available literature, further studies would be necessary to confirm the mechanisms of dye bioremoval with certainty. To this end, continued research into the biological remediation of these dyes will be carried out.

## 4 Conclusions

*P. aeruginosa* was used with different success in the biodecolorization of different textile dyes. While the removal of cationic ST was reduced at higher concentrations, the decolorization of the anionic IC dye was almost quantitative within 8 h at higher initial concentrations, enhancing the removal efficiency and decolorization rate. IC biodecolorization was attained under moderate conditions of time, temperature, pH, and agitation, showing the potential of the selected bacterial strain for practical applications.

This different effectiveness of *P. aeruginosa* in the bioremediation emphasizes the role of dye structure and its surface charge in removal efficiency, suggesting different removal mechanisms for each dye. The enzymatic degradation of IC is supported by two factors, the presence of sulfonated groups in its structure, since *P. aeruginosa* is capable of cleaving C–S bonds, and the substrate concentration-dependent behavior, which regulates the effective induction of the enzymes.

On the other hand, the presence of a precipitate of strongly colored cells and practically no effect on decolorization when incubated in different nutritional inputs or different oxygen availability (static vs. aerated), along with the positive surface charge, reinforces that the removal mechanism of the ST dye was via biosorption.

In addition, this study showed that the bioremoval of both dyes was significantly influenced by agitation, regardless of the mechanism involved, further highlighting as a crucial operational parameter for optimizing bioremediation processes.

By clarifying these mechanistic aspects, this study provides a more comprehensive understanding of how different dye types interact with bacterial cells, offering guidance for future research and industrial applications in wastewater treatment. Furthermore, they reinforce the potential of bacteria-based treatments as a sustainable and cost-effective alternative for textile effluent remediation, contributing to the advancement of eco-friendly biotechnological solutions.

## Supporting Information Available

Chemical structure and main properties of indigo carmine (IC) and (b) safranine-T (ST) dyes (PDF)
