# Supplementary material for: Harnessing Pseudomonas aeruginosa for Bioremediation: Comparative Study on the Removal of Indigo Carmine and Safranine-T Textile Dyes
Source: ACS Omega. 2025 Apr 9;10(15):14676–86. doi: 10.1021/acsomega.4c07948 (PMC12019516; doi:10.1021/acsomega.4c07948)
Supplement: Supplementary file 1 — ao4c07948_si_001.pdf [file ao4c07948_si_001.pdf]

Harnessing *Pseudomonas aeruginosa* for bioremediation: Comparative study on removal of Indigo Carmine and Safranine-T textile dyes

Magali Teresinha Ritter[1,2], Maria Eliza Nagel-Hassemer[1], Ricardo Mazzon[3], Amanda Silva Hecktheuer[3] and María Ángeles Lobo-Recio[4,5]*

[1] Department of Environmental Engineering, Federal University of Santa Catarina (UFSC), Campus Reitor João David Ferreira Lima, 88.040-900, Florianópolis, SC, Brazil

[2] Department of Applied Chemistry, Autonomus University of Madrid (UAM), Francisco Tomás y Valiente Street, 2, University City of Cantoblanco, 28049, Madrid, Spain

[3] Department of Microbiology, Immunology and Parasitology, UFSC, Campus Reitor João David Ferreira Lima, 88.040-900, Florianópolis, SC, Brazil

[4] Graduate Programm on Environmental Engineering, UFSC, Campus Reitor João David Ferreira Lima, 88.040-900, Florianópolis, SC, Brazil

[5] Department of Energy and Sustainability, UFSC, Campus Araranguá, Rod. Gov. Jorge Lacerda, 3201, Jardim das Avenidas, 88.906-072, Araranguá, SC, Brazil

*Corresponding author. magali.ritter@posgrad.ufsc.br (Magali Teresinha Ritter)

Supporting Information

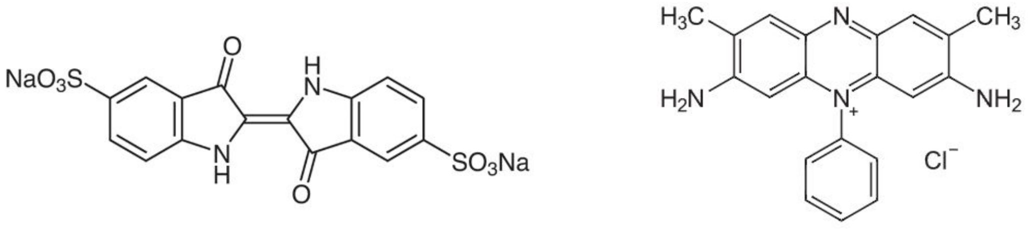

(a) (b)

Figure S1. Chemical structure of (a) Indigo Carmine (IC) and (b) Safranine-T (ST) dyes.

Table S1. Main properties of Indigo Carmine (IC) and Safranine-T (ST) textile dyes.

Dyes	IC	ST
Chemical form	$C_{16}H_8N_2Na_2O_8S_2$	$C_{20}H_{19}ClN_4$
IUPAC	5,5'-indigodisulfonic acid sodium salt	3,7-dimethyl-10-phenylphenazin-10-ium-2,8-diamine;chloride
CAS number	860-22-0	477-73-6
Molecular weight (g mol^{-1})	466.4	350.8
UV absorption (nm)	610	520
